# Factors associated with mortality in older patients sustaining pelvic or acetabular fractures

**DOI:** 10.1007/s00402-021-03873-5

**Published:** 2021-04-03

**Authors:** Anna Harrison, Alejandro Ordas-Bayon, Mukai Chimutengwende-Gordon, Mary Fortune, Daud Chou, Peter Hull, Andrew Carrothers, Jaikirty Rawal

**Affiliations:** 1 Division of Trauma and Orthopaedic Surgery, Addenbrooke’s Hospital, Cambridge University Hospitals NHS Foundation Trust, Cambridge, UK; 2 School of Clinical Medicine, University of Cambridge, Cambridge, UK

**Keywords:** Acetabular fracture, Pelvic fracture, Geriatric trauma, Mortality

## Abstract

**Introduction:**

This study aimed to investigate potential factors, including delay to surgical stabilisation, affecting mortality in older patients sustaining pelvic or acetabular (PA) fractures.

**Materials and methods:**

A retrospective review of the Trauma Audit and Research Network (TARN) database was performed to identify older patients (aged 65 and over) sustaining PA fractures treated surgically in a UK Major Trauma Centre (MTC) between 2015 and 2019. Chi-squared and Fisher tests were used to compare 1-year mortality rates following operative intervention between patients treated within 72 h and after 72 h. Kaplan–Meier curves were used to visualise survival probability; significant predictors of survival were found using Cox proportional hazard models.

**Results:**

Of 564 older patients with PA fractures, 70 met the inclusion criteria. The mean age was 76.1 years. The overall 1-year mortality rate was 20%. When patients were grouped by time to surgery (fracture fixation within or greater than 72 h), there was no statistically significant difference in 1-year mortality. Patients whose surgery was delayed more than 72 h were more likely to have longer hospital stays (*p* = 0.002) or to have suffered from polytrauma (*p* = 0.025). Age, Charlson Co-morbidities Index (CCI) and pre-op mobility status were associated with statistically significant differences in overall mortality. The same factors were associated with a significantly increased hazard of death in the multivariate Cox proportional hazards model. Patient gender, mechanism of injury, Injury Severity Score (ISS) > 15 and head injury were not significant predictors of mortality.

**Conclusion:**

Surgical intervention within 72 h of injury did not result in decreased mortality in older patients with PA fractures. The 1-year mortality rate between older PA fractures and hip fractures was comparable. Consideration should be given to a combined multidisciplinary approach between orthogeriatric and expert PA surgeons for these patients.

## Introduction

The number of people aged 65 and over in the UK is projected to rise by over 40% in the next 25 years, to more than 17 million [1]. Additionally, the demographics of the polytrauma patient have changed over time; most notably with mean age, increased from 36 in 1990 to 54 years in 2013 [2]. In 2013, the most common mechanism of injury resulting in polytrauma in England and Wales was a fall from standing height (40%) compared to road traffic collisions (RTC) (60%) in 1990. With the contribution of isolated fragility fractures and polytrauma in the older patient there is a predicted significant increase in the incidence of pelvic and acetabular (PA) fractures in the older population [2].

Similar to hip fractures, PA fractures through physiological derangement and pain may lead to prolonged immobility, which has a demonstrable effect on mortality in the elderly [3]. 1-year mortality rates for conservatively and surgically managed older patients sustaining PA fractures range from 16 to 33% for acetabular fractures [4, 5] to 27% for pelvic fractures [6]. While the relationship between early surgical intervention and mortality reduction in proximal and distal femur fractures is well established [7, 8], there is no clear consensus concerning PA fractures. The British Orthopaedic Association Standards for Trauma and Orthopaedics (BOAST) guidelines recommend surgery within 72 h of injury for pelvic fractures [9], but clear evidence and its association between mortality rates does not exist.

The aim of this study is to assess whether a delay in time to surgery following PA fractures in the older patient affects mortality rates. The primary outcome is to determine mortality rates at 30 days, 3 months and 1-year in patients who have undergone surgery within 72 h of injury, and those who have undergone surgery after 72 h of injury. Secondary objectives are to determine if a relationship exists between survival and independent factors such as duration of time from injury to surgery, age, Charlson Comorbities Index (CCI), pre-op mobility status and polytrauma [defined by an Injury Severity Score (ISS) [10] > 15 points].

## Patients and methods

### Data collection

A retrospective evaluation of the Trauma Audit Research Network (TARN) database was conducted. TARN collects data in England and Wales on trauma patients admitted to hospital for greater than or equal to 72 h, who require critical care resources or who die from their injuries. The TARN search included patients aged 65 and older who were admitted to our hospital and were treated with a PA fracture from 2015 to 2019. This time frame was selected in order to be able to match results and obtain secondary variables with the hospital medical records software (Epic Software Systems, WI, USA) implemented in our centre in 2015. Patients for whom 1-year data were not available at the time of data collection were excluded. Inclusion and exclusion criteria are summarised in Table 1.

**Table 1:** Inclusion and exclusion criteria

Inclusion	Exclusion
Aged 65 and over	Aged less than 65 years old
Pelvic and/or acetabular fracture	Proximal femur fracture
Surgical treatment	Conservative management
At least 1-year follow up	Less than 1-year follow up

### Demographics

Age, gender, date and time of injury, mechanism of injury, ISS, presence of an associated traumatic brain injury (TBI) and pre-op mobility status were obtained from the hospital records. To quantify patient co-morbidities, CCI, which has been validated as a predictor of short and long-term mortality in hospitalised elderly adults [11], was calculated for every patient based on information from clerking notes on admission.

PA fractures were classified using both radiological imaging analysis and surgical operative notes, according to the Young and Burgess Classification for pelvic ring injuries [12]; Judet–Letournel Classification was used for acetabulum fractures [13].

### Time to surgery

Time from injury to surgery was obtained from TARN and was measured in hours. Surgical treatment of the fractures was performed by fellowship-trained PA surgeons. Surgical techniques included open reduction internal fixation (ORIF), percutaneous fixation under fluoroscopy guidance and combined ORIF and total hip arthroplasty (THA). Reason for delay to surgery if applicable was recorded and categorised as: change in management, medical complications, list availability, delay in diagnosis and TBI preventing surgery. Other surgery-related variables obtained were time spent in Intensive Care Unit (ICU) and length of hospital stay (LOS).

### Mortality analysis

The NHS Spine database was used to determine 30-day, 3-month, 1-year and overall mortality. Spine supports the IT infrastructure for health and social care in England, providing a summary of patient demographics including mortality where applicable [14].

### Statistical analysis

Characteristics of patients were compared between two groups: those treated operatively within 72 h and those treated after 72 h. This time cut-off was chosen based on the BOAST guidelines for pelvic fractures. Test for normality was performed using Shapiro–Wilk. Where data was non-normal, a Mann–Whitney *U* test with or without continuity correction was used. Where data were normally distributed, *T* tests were used to compare continuous variables. Chi-squared tests (when the number of observations in each subgroup was at least 5) and Fisher exact tests (when the number of observations in a subgroup was less than 5) were used to compare categorical variables.

The effect of different factors upon the survival time was portrayed using Kaplan–Meier plots and compared using log-rank tests. The hazard ratio for the effect of time delay to surgery upon survival was calculated using multivariate Cox proportional hazards models, with the other factors included in the model in order to control for their effect. For these analysis, time delay was treated both as a dichotomous variable as per BOAST guidelines as well as analysed as a continuous variable, to determine the additive effect of delay without imposing arbitrary thresholds [15]. Age was split into “older” (65–80) and “elderly” (greater than or equal to 80 years old).

In all analyses, a *p* value ≤ 0.05 was considered to be statistically significant. The statistical software package R was utilised for all analyses. The R library “*survival*” was used to perform the survival analyses, and the R library “*survminer*” was used to generate the Kaplan–Meyer plots.

## Results

### Data collection

**Fig. 1:**
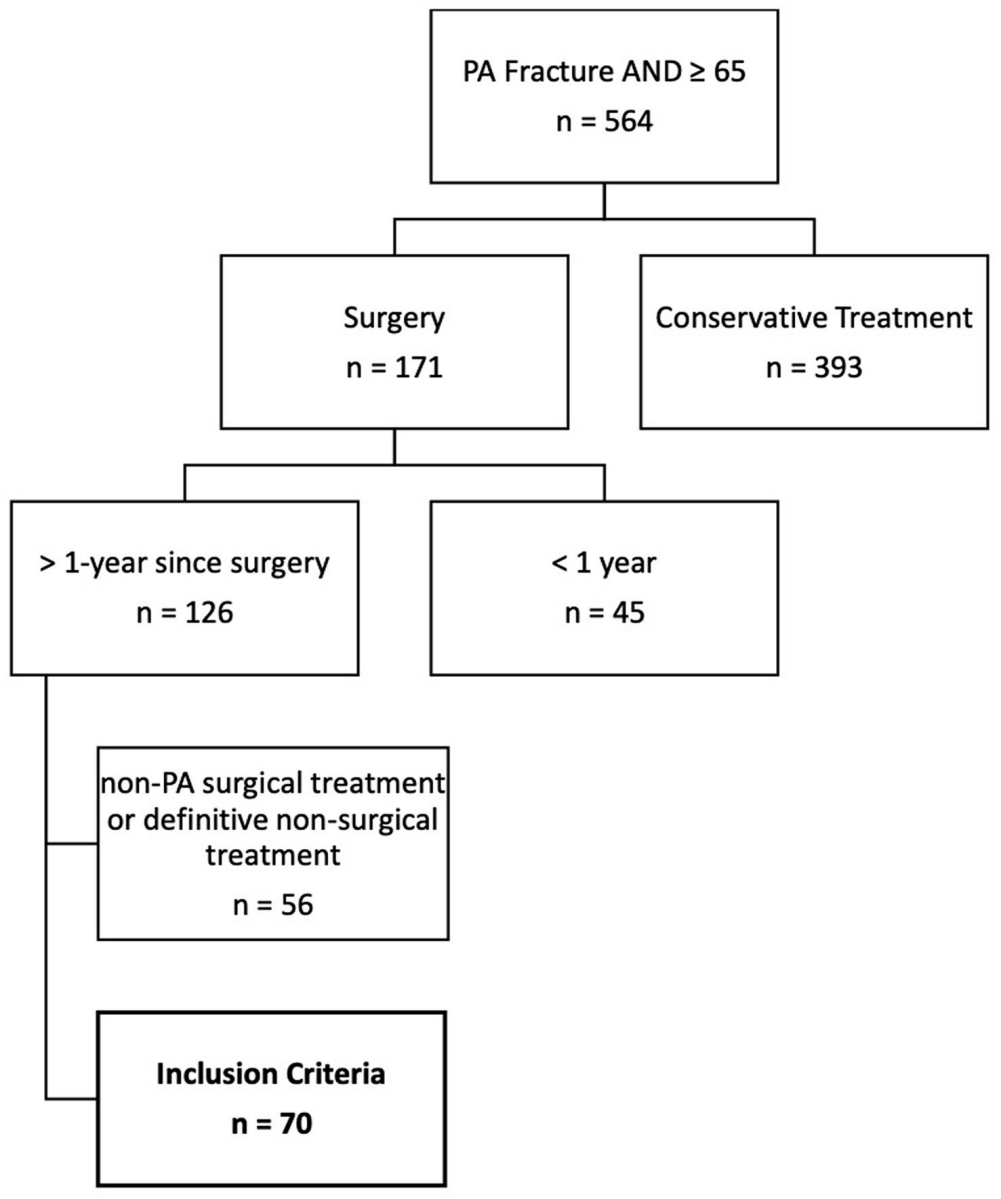
Inclusion/exclusion flow diagram

TARN search identified a total of 564 patients 65 years or older with a PA fracture. Of these, 393 were excluded as non-surgical patients, along with a further 45 patients who did not meet 1-year follow-up criteria. Following cross-correlation with the hospital database, a further 56 patients were excluded who had surgical treatment for non-PA fractures or definitive non-surgical treatment. A total of 70 patients met inclusion criteria (Fig. 1).

### Demographics

43 (61%) patients were males and 27 (39%) were females. The average age was 76 years (65–95). The most common mechanism of injury was fall from standing height (*n* = 39, 56%), with remaining injuries sustained by high-energy mechanisms, mostly following road traffic accidents (*n* = 19, 27%).

The majority of fractures involved the acetabulum (*n* = 53, 76%) with two patients with bilateral acetabular fractures; the remaining fractures were pelvic ring injuries (*n* = 17, 24%). The most common acetabular fracture pattern was Anterior Column Posterior Hemi-transverse (*n* = 17, 32%), followed by Associated Both Columns (*n* = 11, 21%). 19 elementary fractures (36% of acetabular fractures) and 33 (62%) associated fractures were identified. The most common pelvic fracture pattern was lateral compression type 1 (LC-1) (*n* = 10, 59% of pelvic fractures). Further information regarding fracture classification can be found in Table 2.

**Table 2:** Summary of fracture classifications

Acetabular fractures	Total (*n* = 53) (%)
Elementary	
Anterior column	9 (17)
Posterior wall	5 (9)
Transverse	4 (8)
Posterior column	1 (2)
Associated	
Anterior column posterior Hemi-transverse	17 (31)
Associated both columns	11 (21)
Posterior column posterior wall	2 (4)
T shaped	2 (4)
Anterior column posterior wall	1 (2)
Unclassified	1 (2)

Pelvic fractures	Total (*n* = 17) (%)
LC-1	10 (59)
APC-3	3 (18)
VS	3 (18)
APC-2	1 (6)

*LC* lateral compression, *APC* anteroposterior compression, *VS* vertical shear

### Time to surgery

Mean time to surgery was 86.1 h (6.3–333 h). For surgeries that did not occur within the 72 h BOAST guidelines (*n* = 33, 47%), the majority of delay was due to either list availability (*n* = 19, 58%) or medical complications (*n* = 18, 55%). Time spent in Intensive Care Unit (ICU), length of hospital stay (LOS) along with a summary of patient’s characteristics are summarised in Table 3.

**Table 3:** Patient characteristics

Variable	Total (*n* = 70)
Time to surgery, mean (range)	86.1 (6.3–333)
Sex (*n*, %)	
Male	43 (61)
Female	27 (39)
Age, mean (years) (range)	76.1 (65–95.4)
CCI, mean (range)	4.6 (2–11)
Mechanism (*n*, %)	
Fall < 2 m	39 (56)
Fall > 2 m	11 (16)
RTA	19 (27)
Assault	1 (1)
Fracture type	
Acetabular	53 (76)
Pelvic	17 (24)
Polytrauma (*n*, %)	23 (33)
LOS, mean (days) (range)	21.7 (3–157)
LOS post op, mean (days) (range)	18.1 (2.3–157)
LOS ICU, mean (days) (range)	2.84 (0–29)
Pre-op mobility status (*n*, %)	
Independent	38 (54)
With aid	29 (29)
Not specified	3 (4)
Mortality (*n*, %)	
At 30 days	2 (3)
At 90 days	5 (7)
At 1 year	14 (20)

*CCI* Charlson Co-morbidities Index, *LOS* length of hospital stay

### Mortality analysis

Overall 1-year mortality rate was 20% (14 out of 70), with a mortality rate of 17% for those with acetabular fractures (9 out of 53) and 29% for pelvic fractures (5 out of 17) (*p* = 0.444). Patients deceased at 1 year had an average age of 82 years (70–95), CCI of 6.5, and average time to surgery of 111 h; none of them sustained a major head injury.

In the acetabular cohort, the 1-year mortality rate in those treated using ORIF was 29% (5 out of 17), while the 1-year mortality rate in those treated using combined ORIF and THA was 11% (4 out of 36, *p* = 0.126).

### Statistical analysis

40 (57%) patients were operated on within 72 h (mean time 31.2 h, range 6.4–68 h), and 30 (43%) were operated on after 72 h (mean time 160 h, range 80–333 h). Results from comparisons between the two groups are presented in Table 4. Baseline characteristics between the two groups were comparable, with no statistically significant differences in age, gender, mechanism of injury, CCI, pelvic or acetabular fracture or pre-op mobility status.

**Table 4:** Factors associated with mortality in unadjusted comparison

Variable	Operated ≤ 72-h (*n* = 40)	Operated > 72-h (*n* = 30)	*p* value
Male gender (*n*, %)	22 (55)	21 (70)	0.304
Age, mean (years) (range)	75.2 (65–95.4)	77.2 (65.1–88.9)	0.146
CCI, mean (range)	4.4 (2–8)	5.0 (2–11)	0.387
Mechanism (*n*, %)			
Fall < 2 m	21 (52.5)	18 (60)	0.702
Fall > 2 m	6 (15)	5 (17)	1
RTA	13 (32.5)	6 (20)	0.372
Assault	0 (0)	1 (3)	0.429
Fracture type (*n*, %)			
Acetabular	27 (68)	26 (65)	0.117
Pelvis	13 (33)	4 (10)	0.092
Polytrauma (*n*, %)^a^	18 (45)	5 (17)	0.025*
LOS, mean (days) (range)	19.95 (3–157)	24.03 (8–86)	0.002*
LOS post op, mean (days) (range)	18.6 (2.3–157)	17.4 (4.2–82)	0.203
LOS ICU, mean (days) (range)	2.77 (0–25)	2.93 (0–29)	0.727
Pre-op mobility status (*n*, %)			
Independent	21 (52.5)	17 (57)	0.973
With aid	16 (40)	13 (43)	0.972
Not specified	3 (7.5)	0 (0)	0.255
Mortality (*n*, %)			
At 30 days	1 (2.5)	1 (3)	1
At 90 days	3 (7.5)	2 (7)	1
At 1 year	8 (20)	6 (20)	1

*Statistically significant results

^a^Polytrauma defined as ISS > 15

There was a statistically significant difference in length of overall hospital stay, with a mean of 19.9 days (0–25) for those operated on within 72 h, and a mean of 24 days (0–29) for those operated on after 72 h (*p* = 0.002). There was also a statistically significant difference in number of polytrauma patients operated on within 72 h (*n* = 18, 45%), versus the number of polytrauma patients operated on after 72 h (*n* = 5, 17%). We found no statistically significant differences in 30-day, 90-day or 1-year mortality between the two groups.

**Fig. 2:**
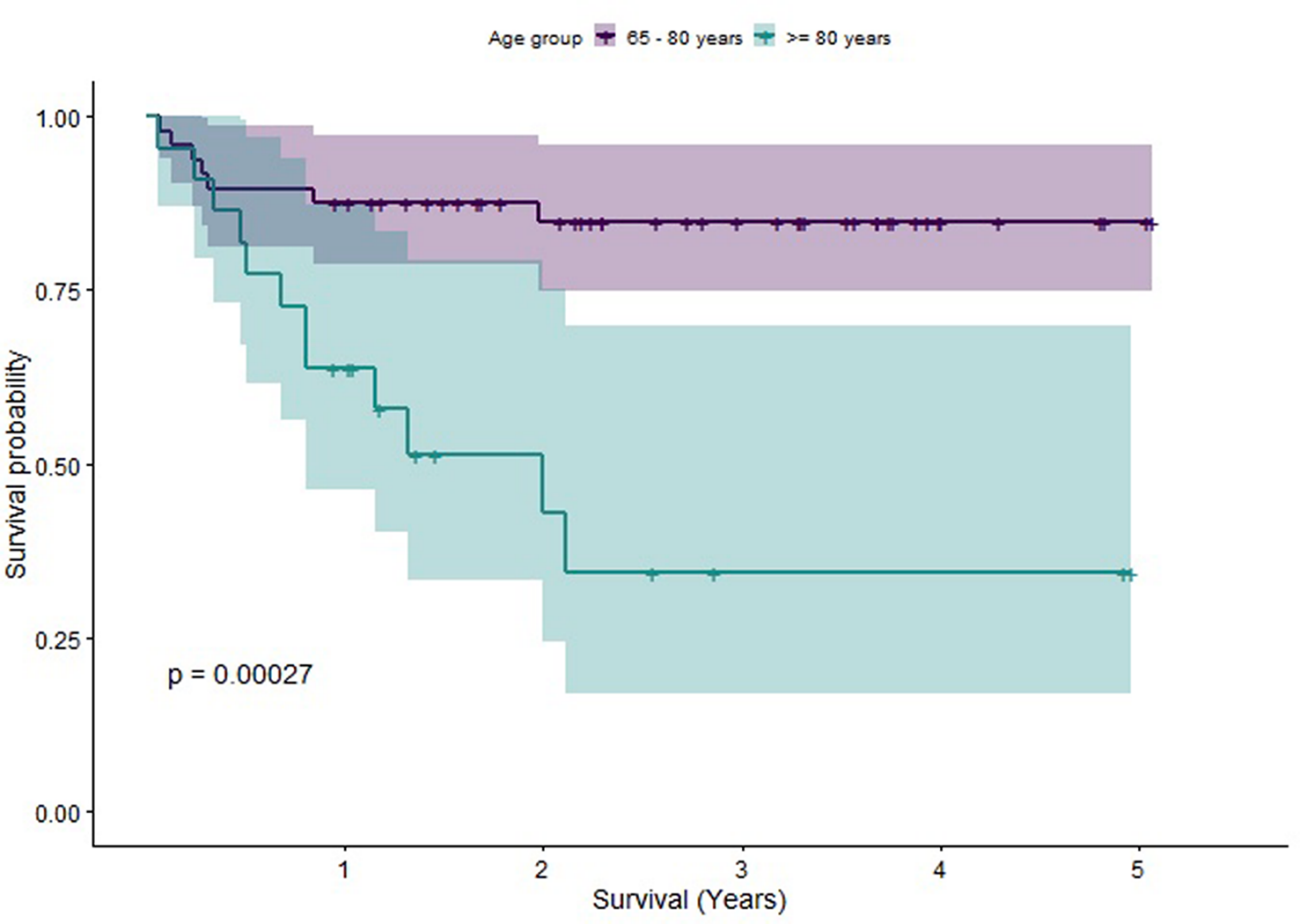
Graph showing the Kaplan–Meier Survival curve for patients grouped into older and elderly

**Fig. 3:**
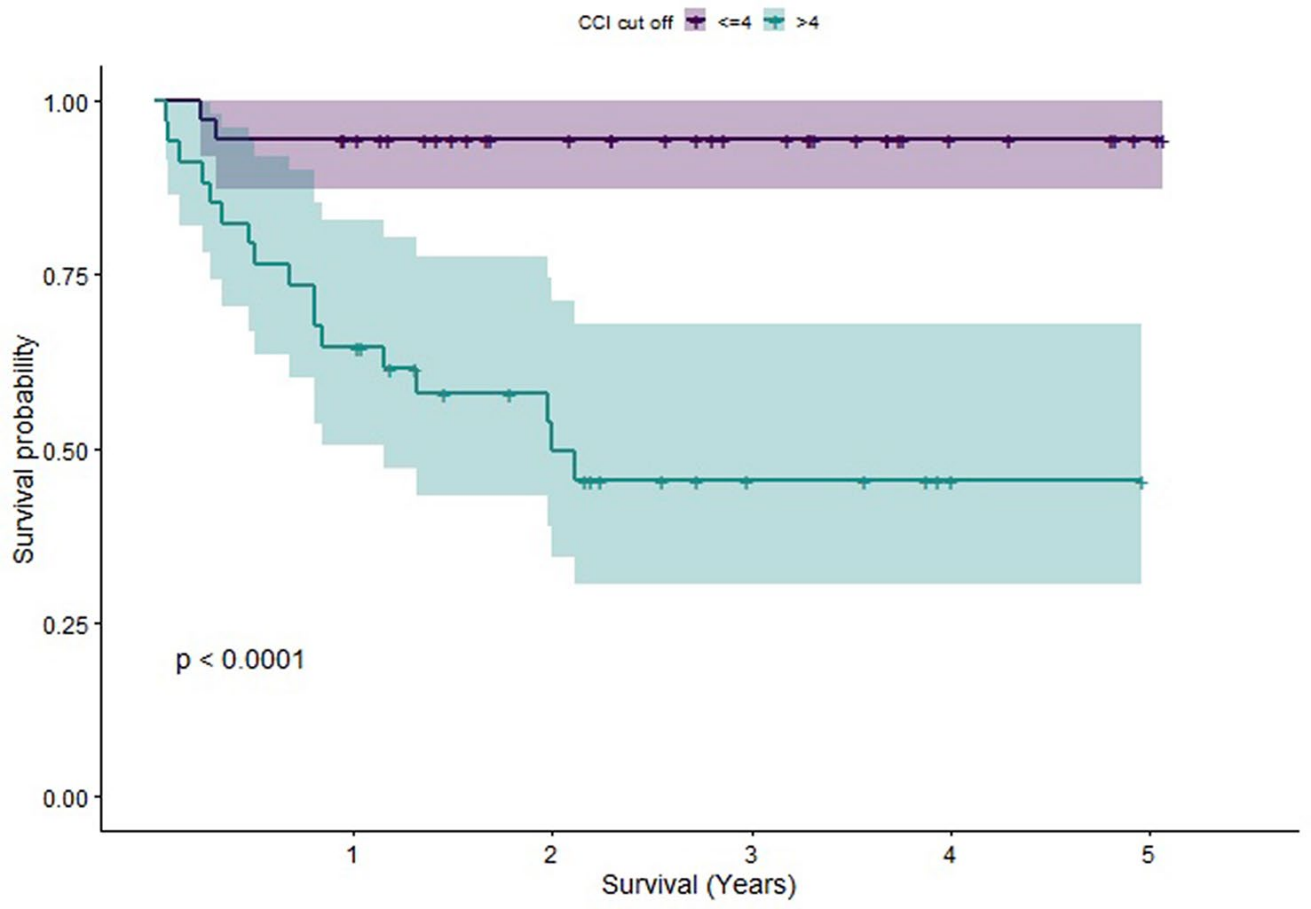
Graph showing the Kaplan–Meier Survival curve for patients split by CCI

**Fig. 4:**
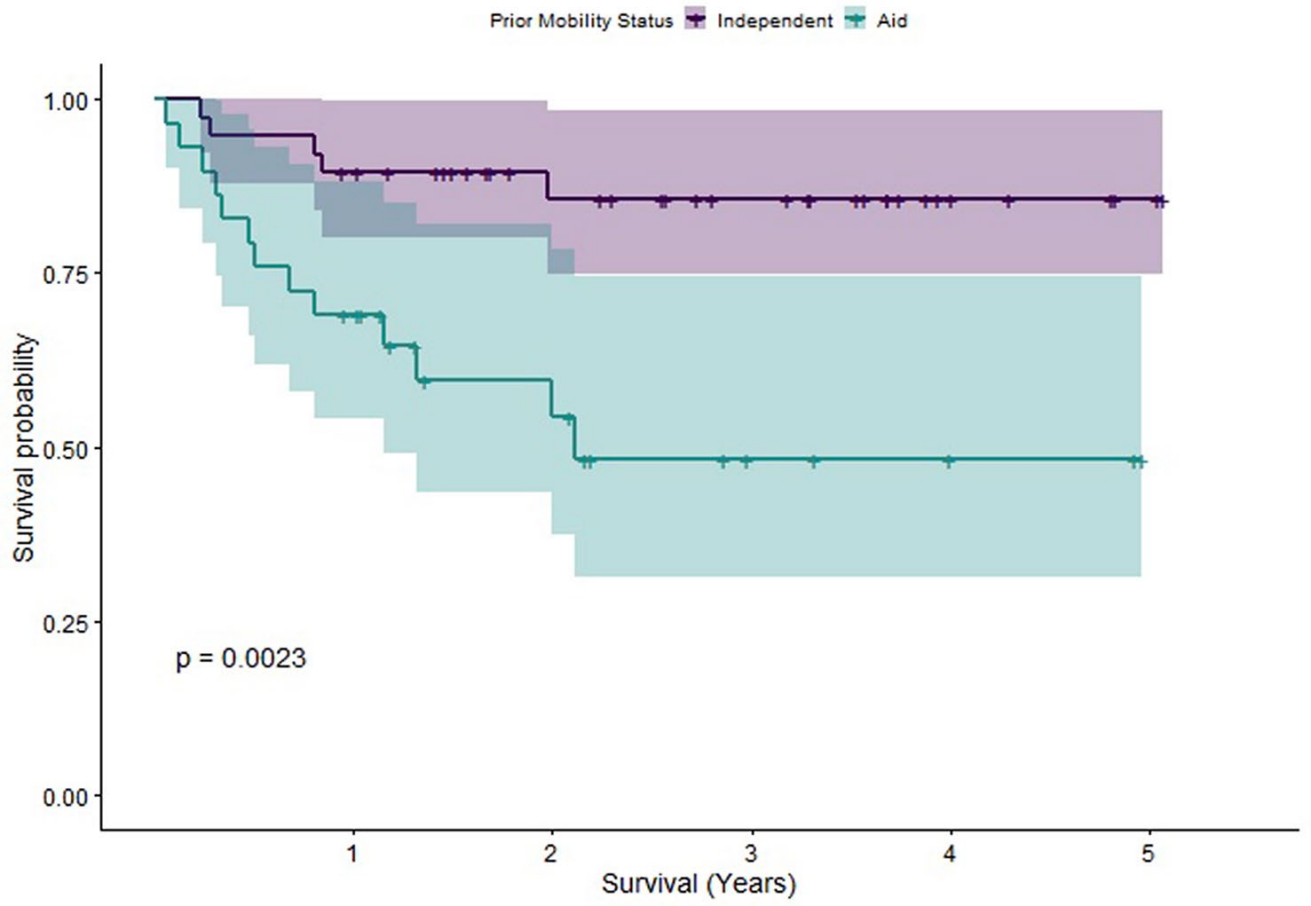
Graph showing the Kaplan–Meier Survival curve for patients grouped according to pre-op mobility status

**Fig. 5:**
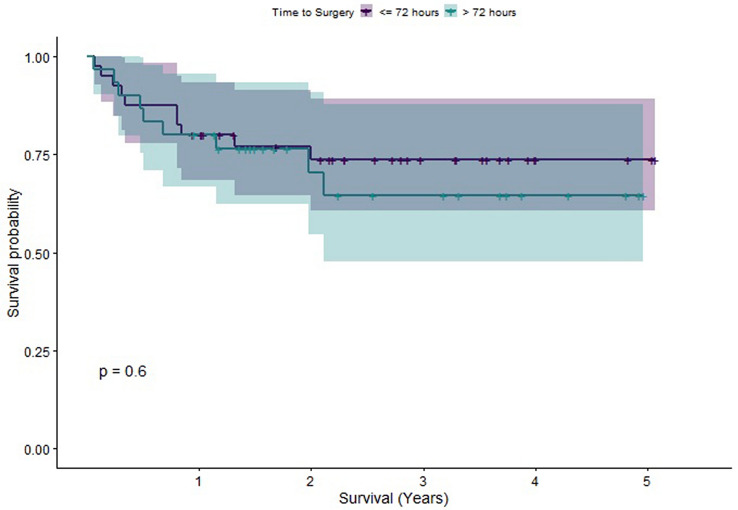
Graph showing the Kaplan–Meier Survival curve for patients grouped according to time to surgery below and above 72 h

In order to understand the individual effects of different factors upon survival, we computed Kaplan–Meier estimators. Log-rank tests were performed to compare these estimators. Statistically significant differences in survival were found between patients grouped as older (65–80) versus those grouped as elderly (older than 80), between patients with a CCI less than or equal to 4 compared to those with CCI greater than 4 (a score of 4 was chosen for the cut-off due to it being the median CCI) and between patients whose pre-op mobility status was assessed as being independent versus those requiring walking aids. The Kaplan–Meier Survivorship plots for these comparisons are illustrated in Figs. 2, 3, 4. When CCI was considered, with points for age excluded, statistically significant differences in survival were also found. However, no statistically significant difference in survival was found when patients were grouped by time to fracture fixation less than 72 h versus time to fracture fixation greater than 72 h (see Fig. 5 for the Kaplan–Meier survivorship plot). In addition, no significant differences in survival were found when patients were grouped by presence of polytrauma, gender, presence of a TBI or pelvic versus acetabular fractures.

A multivariate Cox proportional hazards model was used to find the effect of time delay to surgery on survival, controlling for the presence of baseline factors, and again found no significant difference in mortality rate between patients treated within 72 h and those treated after 72 h.

In order to investigate the additive effect of time delay to surgery upon the hazard rate, without imposing arbitrary thresholds, a second multivariate Cox proportional hazards model was used. The results of this model are given in Table 5. There was no evidence to show that increasing time to surgery was a predictor of mortality. Increasing age, CCI and pre-op mobility status were all significant predictors of mortality. Sex, mechanism of injury and TBI were not significant predictors of mortality. Polytrauma, defined as ISS greater than 15, showed a borderline significance.

**Table 5:** Results of multivariate Cox regression analysis

	HR	*p* value	CI (95%)
Male gender	0.60	0.352	1.21–1.75
Age	1.15	0.006*	1.05–1.27
CCI	1.77	< 0.001*	1.28–2.43
Time to surgery	1.00	0.880	0.99–1.00
MOI	1.00	0.991	0.40–2.55
Polytrauma	4.80	0.061*	0.92–24.7
Pre-op mobility status	2.49	0.038*	1.05–5.90
Head Injury	1.62	0.654	0.20–13.4

*Statistically significant results

## Discussion

In comparison to hip fractures in the elderly, the volume of literature for geriatric PA fractures is diminutive. Studies examine the benefits of surgical versus non-operative intervention in these patients, as well as analysis of the best surgical approach in PA fractures in the elderly [4, 16]. However, there exists a paucity of literature regarding timing of PA surgery post trauma and its impact on the patient. In the hip fracture population, time to surgical intervention has been shown to be a significant factor in predicting mortality in geriatric patients with hip fractures [7, 8] with surgical intervention for hip fractures in the elderly required within 36 h of injury in the UK to qualify for Best Practice Tariffs (BPT) [17].

BOAST guidelines in the UK suggest that patients sustaining a pelvic fracture should be operated on within 72 h of physiological stability [9]; isolated acetabular fractures are excluded from this guideline, with old guidelines suggesting fixation of these fractures within 5 days of injury [18]. However, there is a lack of literature to support these guidelines, in contrast to the general recognition within the surgical community that time to surgery in fragility hip fracture is significantly associated with increased risk of death [7, 8]. Hip fracture management also benefits from well-established pathways, non-centralised surgery and an increase in priority and status through national health policies. PA fractures are usually centralised to MTCs requiring a referral and patient transfer process that creates a time pressure of its own.

Across the age spectrum, no association has been demonstrated between time to PA surgery and mortality [19]. To the best of our knowledge, a recent study by Glogovac et al. [20] is the only paper that investigates the relationship between time to surgery and mortality rates in geriatric patients with acetabular fractures; pelvic fractures were not included. Glogovac et al. grouped patients according to those treated operatively within 48 h of injury and after 48 h of injury, based on the hip fracture guidelines in the United States, and ran a secondary analysis based on a 72 h threshold. They found no relationship between mortality rates at 1 year in either analysis; no assessment was made of time delay to surgery as a continuous variable. In addition, they did not find that patients who had a delay to surgery of greater than 48 h were more likely to have a longer hospital stay.

Our study results show that not only was the cut-off of 72 h, as per BOAST guidelines in the UK, not associated with increased mortality, but also that when time was considered as a continuous variable in the multivariate Cox proportional hazards model, no increase in overall mortality risk was associated with surgical time delay.

Although our study has found no difference of mortality, delay to surgery for acetabular fractures is known to be associated with poorer radiographic and functional outcomes as it can lead to more difficulty in achieving an anatomical fracture reduction [21]; therefore, we support the principle that PA surgery should be undertaken in a timely manner where possible.

While there was no statistical difference in mortality rates between the two groups, the length of hospital stay in those operated on after 72 h was significantly greater (by 4.1 days). The Trauma Network lead time, whilst waiting for transfer of patients to the Major trauma centre, will contribute to the association of ‘delayed surgery’. Prolonged LOS is associated with an increase in hospital-acquired infections, financial cost, disruption of patient flow and resultant bed shortages. In an increasingly resource constrained National Health Service, a reduction in LOS is of significant importance.

This study illustrates a significant difference between the number of polytrauma patients operated on within 72 h and those operated on after 72 h. Contrary to what might be expected, a greater number of these patients were operated on within 72 h (*n* = 18) compared to five after 72 h. This could be due to the very low number of TBIs in this cohort (2 of 23) which can lead to a delay in physiological optimisation to allow surgery.

Our study has illustrated a difference in significance of timing of surgery between older/elderly PA surgery and older/elderly hip fractures. However, the 20% 1-year mortality rate for the PA fracture population is comparable to that of hip fractures; literature suggests a range between 16 and 33% [4, 5] in the PA population at 1 year, versus 15 and 36% [22] in the older/elderly hip fracture population.

A higher mortality rate was found in the ORIF cohort (29%) versus the combined ORIF and THA (11%) for the acetabular sub-group. While the *p* value of 0.126 does not suggest that this difference in mortality rates is significant, it does suggest that there was not an increase in mortality in the “fix and replace” cohort, which has traditionally been seen as a technique that poses greater risk to the patient.

The Blue Book [23], a joint publication by the British Orthopaedic Association and British Geriatric Society, encourages the co-operation between orthopaedic surgeons and geriatricians in the recognition and management of hip fractures in the UK. The introduction of Fracture Liaison Services (FLS) has resulted in considerable mortality benefits, with 30-day mortality falling by 7.6% per year in the 4 years after the introduction of the National Hip Fracture Database compared to a 1.8% per year decrease in in the 4 years preceding its introduction [24]. Additionally, in England the appropriate management of hip fractures in the elderly population attracts a best practice tariff incentive (BPT) [17]. However, for the older/elderly PA population, no such incentive exists, despite comparable mortality rates.

This study found an association between increased age, pre-existing co-morbidities and pre-op mobility status on overall mortality. These factors form 3 of the 7 criteria in the PRISMA-7 questionnaire, recommended by NHS England as a tool for identifying frailty. Older people with frailty who need to undergo surgery can have less successful outcomes if the frailty has not been identified prior to the operation [25]. Recent evidence suggests that sarcopenia, a physical measure of frailty, could also be associated with higher 1-year mortality in older/elderly patients with acetabular fractures [26, 27].

In view of patient outcomes, and the significant improvement in 30-day mortality shown since the introduction of the National Hip Fracture Database, we would consider stricter adherence to an integrated orthogeriatric pathway for PA fractures in the older population. While patients with hip fractures have recently been the focus of such collaboration, the PA fracture population has largely been overlooked. This multidisciplinary approach could enable more streamlined management of the physical injury, optimisation of comorbidities and rehabilitation, including measures to prevent future further injury; overall allowing for better outcomes for these patients.

Limitations of this study include the small sample size (limited by the conception of the hospital’s electronic database in 2015), the retrospective design and lack of postoperative functional outcomes as another measure of enduring morbidity post-surgical intervention.

## Conclusion

In conclusion, our study found that time delay to surgery following pelvic or acetabular fracture in older patients does not affect 1-year mortality rates, in contrast to the decreased mortality associated with early surgical intervention in elderly patients with neck of femur fractures. This evidence leads us to question the mortality benefits of definitive time criteria on surgical management of older/elderly patients with PA fractures. However, there are the described associated benefits for total length of hospital stay, both clinical and financial, and it is hard to corroborate evidence quantifying the compassionate arguments for early fracture stabilization and analgesic effect. Therefore, the authors are in support of the principle behind the 72 h goal of achieving surgical stability for his cohort of patients, but the justification may not be an effect on 1-year mortality, but rather an effect on length of stay. Increased mortality was independently associated with increasing age, co-morbidities and previous mobility status. Further work is required to consider a more integrated orthogeriatric care pathway for this patient population.

## References

[1] ONS (2019) National Population Projections: 2018 based. https://www.ons.gov.uk/peoplepopulationandcommunity/populationandmigration/populationprojections/bulletins/nationalpopulationprojections/2018based. Accessed 26 May 2020

[2] Kehoe A, Smith JE, Edwards A, Yates D, Lecky F (2015). The changing face of major trauma in the UK. Emerg Med J.

[3] Harper CM, Lyles YM (1988). Physiology and complications of bed rest. J Am Geriatr Soc.

[4] Firoozabadi R, Cross WW, Krieg JC, Routt MLC (2017). Acetabular fractures in the senior population- epidemiology, mortality and treatments. Arch Bone Jt Surg.

[5] Gary JL, Paryavi E, O’Toole RV, Gibbons SD, Starr AJ, Weaver MJ, Morgan JH, Ryan SP (2015). Effect of surgical treatment on mortality after acetabular fracture in the elderly: a multicenter study of 454 patients. J Orthop Trauma.

[6] Banierink H, Ten Duis K, de Vries R, Wendt K, Heineman E, Reininga I, IJpma F (2019). Pelvic ring injury in the elderly: fragile patients with substantial mortality rates and long-term physical impairment. PLoS ONE.

[7] Rosso F, Dettoni F, Bonasia DE, Olivero F, Mattei L, Bruzzone M, Marmotti A, Rossi R (2016). Prognostic factors for mortality after hip fracture: operation within 48 hours is mandatory. Injury.

[8] Moja L, Piatti A, Pecoraro V, Ricci C, Virgili G, Salanti G, Germagnoli L, Liberati A, Banfi G (2012). Timing matters in hip fracture surgery: patients operated within 48 hours have better outcomes. A meta-analysis and meta-regression of over 190,000 patients. PLoS ONE.

[9] BOAST (2018) The management of patients with pelvic fractures. https://www.boa.ac.uk/resources/boast-3-pdf.html. Accessed 26 May 2020

[10] Linn S (1995). The injury severity score—importance and uses. Ann Epidemiol.

[11] Frenkel WJ, Jongerius EJ, Mandjes-van Uitert MJ, van Munster BC, de Rooij SE (2014). Validation of the Charlson comorbidity index in acutely hospitalized elderly adults: a prospective cohort study. J Am Geriatr Soc.

[12] Alton TB, Gee AO (2014). Classifications in brief: Young and Burgess classification of pelvic ring injuries. Clin Orthop Relat Res.

[13] Alton TB, Gee AO (2014). Classifications in brief: Letournel classification for acetabular fractures. Clin Orthop Relat Res.

[14] NHS Spine database (2020). https://digital.nhs.uk/services/spine#top. Accessed 8 Jun 2020

[15] Royston P, Altman D, Sauerbrei W (2006). Dichotomizing continuous predictors in multiple regression: a bad idea. Stat Med.

[16] Hanschen M, Pesch S, Huber-Wagner S, Biberthaler P (2017). Management of acetabular fractures in the geriatric patient. SICOT J.

[17] NHS England 2019/20 (2019) National tariff payment system—guidance on best practice tariffs. https://improvement.nhs.uk/resources/national-tariff-1920-consultation/#annexes. Accessed 26 May 2020

[18] BOAST (2008) BOAST 3: Pelvic and acetabular fracture management. https://www.juniorbones.com/uploads/2/5/8/8/25885124/boast_3_-_pelvic_and_acetabular_fracture_management.pdf. Accessed 8 Jul 2020

[19] Devaney GL, Bulman J, King KL, Balogh ZJ (2020). Time to definitive fixation of pelvic and acetabular fractures. J Trauma Acute Care Surg.

[20] Glogovac G, Le TT, Archdeacon MT (2020). Time to surgery and patient mortality in geriatric acetabular fractures. J Orthop Trauma.

[21] Madhu R, Kotnis R, Al-Mousawi A, Barlow N, Deo S, Worlock P, Willett K (2006). Outcome of surgery for reconstruction of fractures of the acetabulum. The time dependent effect of delay. J Bone Jt Surg Br.

[22] Morri M, Ambrosi E, Chiari P, Magli AO, Gazineo D, D'Alessandro F, Forni C (2019). One-year mortality after hip fracture surgery and prognostic factors: a prospective cohort study. Sci Rep.

[23] BOA (2007) The care of patients with fragility fractures. https://www.bgs.org.uk/sites/default/files/content/attachment/2018-05-02/Blue%20Book%20on%20fragility%20fracture%20care.pdf. Accessed 27 May 2020

[24] Neuburger J, Currie C, Wakeman R, Tsang C, Plant F, De Stavola B, Cromwell DA, van der Meulen J (2015). The impact of a national clinician-led audit initiative on care and mortality after hip fracture in England: an external evaluation using time trends in non-audit data. Med Care.

[25] Identifying frailty (2020). https://www.england.nhs.uk/ourwork/clinical-policy/older-people/frailty/frailty-risk-identification/. Accessed 25 Jun 2020

[26] Wong RMY, Wong H, Zhang N, Chow SKH, Chau WW, Wang J, Chim YN, Leung KS, Cheung WH (2019). The relationship between sarcopenia and fragility fracture-a systematic review. Osteoporos Int.

[27] Deren ME, Babu J, Cohen EM, Machan J, Born CT, Hayda R (2017). Increased mortality in elderly patients with sarcopenia and acetabular fractures. J Bone Jt Surg Am.

